# Low-Dose Decitabine Inhibits Cytotoxic T Lymphocytes-Mediated Platelet Destruction *via* Modulating PD-1 Methylation in Immune Thrombocytopenia

**DOI:** 10.3389/fimmu.2021.630693

**Published:** 2021-02-17

**Authors:** Panpan Han, Tianshu Yu, Yu Hou, Yajing Zhao, Yang Liu, Yunqi Sun, Haoyi Wang, Pengcheng Xu, Guosheng Li, Tao Sun, Xiang Hu, Xinguang Liu, Lizhen Li, Jun Peng, Hai Zhou, Ming Hou

**Affiliations:** ^1^Department of Hematology, Qilu Hospital, Cheeloo College of Medicine, Shandong University, Jinan, China; ^2^Shandong Key Laboratory of Immunohematology, Qilu Hospital, Cheeloo College of Medicine, Shandong University, Jinan, China; ^3^Shandong Provincial Clinical Medicine Research Center for Hematology, Qilu Hospital, Cheeloo College of Medicine, Shandong University, Jinan, China

**Keywords:** immune thrombocytopenia, decitabine, cytotoxic T lymphocytes, PD-1, PD-L1

## Abstract

Cytotoxic T lymphocytes (CTLs)-mediated platelet destruction plays an important role in the pathogenesis of primary immune thrombocytopenia (ITP). The programmed cell death protein 1 (PD-1) signaling can turn off autoreactive T cells and induce peripheral tolerance. Herein, we found that the expression of PD-1 and its ligand PD-L1 on CD8^+^ T cells from ITP patients was decreased. Activating PD-1 pathway by PD-L1-Fc fusion protein inhibited CTLs-mediated platelet destruction in ITP *in vitro*. PD-1 promoter hypermethylation in CD8^+^ T cells was found in ITP patients, resulting in decreased PD-1 expression. The demethylating agent decitabine at a low dose was proved to restore the methylation level and expression of PD-1 on CD8^+^ T cells and reduce the cytotoxicity of CTLs of ITP patients. The phosphorylation levels of phosphatidylinositol 3-kinase (PI3K) and AKT in CD8^+^ T cells were significantly downregulated by low-dose decitabine. Furthermore, blocking PD-1 could counteract the effect of low-dose decitabine on CTLs from ITP patients. Therefore, our data suggest that the aberrant PD-1/PD-L1 pathway is involved in the pathophysiology of ITP and enhancing PD-1/PD-L1 signaling is a promising therapeutic approach for ITP management. Our results reveal the immunomodulatory mechanism of low-dose decitabine in ITP by inhibiting CTLs cytotoxicity to autologous platelets through PD-1 pathway.

## Introduction

Primary immune thrombocytopenia (ITP) is an acquired autoimmune disease characterized by immune-mediated platelet destruction and impaired platelet production (1–4). In addition to platelet autoantibodies, abnormal T cell immunity, especially CD8^+^ cytotoxic T lymphocytes (CTLs) play a vital role in the pathogenesis of ITP (5–8). CTLs have been demonstrated to cause direct platelet lysis in patients with active ITP (9).

Programmed cell death protein 1 (PD-1), a member of the CD28/CTLA-4 family, is inducibly expressed on peripheral T cells, B cells, natural killer T cells, monocytes and some dendritic cells upon their activation (10, 11). In combination with T cell receptor (TCR) signal, PD-1/PD-L1 signaling pathway is critical in turning off autoreactive T cells and reducing CTLs cytotoxicity to target cells. Aberrant PD-1/PD-L1 signaling results in the breakout of peripheral tolerance and is an important contributor to autoimmune diseases, such as rheumatoid arthritis (RA), systemic lupus erythematosus (SLE), and encephalomyelitis (12–14). Previous studies have shown that PD-1 and PD-L1 expression on peripheral blood mononuclear cells (PBMCs) and CD4^+^ T cells were lower in ITP patients (15, 16). *In vitro* studies have proved that PD-L1-Fc recombinant fusion protein (PD-L1-Fc, linking the extracellular domains of PD-1) increased T cell apoptosis and inhibited the activation and proliferation of T cells in ITP, suggesting the important role of PD-1 pathway in the pathogenesis of ITP (17).

Decitabine, a hypomethylating agent (HMA), has been used in the treatment of myelodysplastic syndrome (MDS). Recently, low-dose decitabine was shown to promote megakaryocyte maturation *in vitro* and platelet release *in vivo*, which was closely related to the demethylating effect of decitabine (18). Our previous studies showed that low-dose decitabine significantly increased the number of mature polyploidy megakaryocytes and had a clinical long-term efficacy that could not be annotated by the effect of decitabine on promoting platelet release from these megakaryocytes (19, 20). It has been found that hypomethylating agents resulted in the demethylation of the PD-1 CpG island and increased PD-1 expression in MDS (21).

In this study, decreased PD-1 and PD-L1 expression on CD8^+^ T cells was found in active ITP patients. PD-L1-Fc was proven to successfully inhibit the cytotoxicity of CTLs. PD-1 promoter methylation level in CD8^+^ T cells was increased and could be restored by low-dose decitabine. We also demonstrated that low-dose decitabine reduced CTLs-mediated platelet destruction, which could be partly counteracted by PD-1 blockade. Our results revealed the immunomodulatory mechanism of low-dose decitabine in ITP by inhibiting CTLs cytotoxicity through PD-1 pathway.

## Methods

### Patients and Controls

Sixty-five patients with active ITP (34 females and 31 males; age range 16–81 years, median 47 years) were consented in the study between May 2016 and January 2021 in the Department of Hematology, Qilu Hospital, Shandong University. Fourteen ITP patients were enrolled and received decitabine previously described (20). All patients fulfilled the clinical diagnostic criteria for ITP as previously published (22) and were free from any previous therapy for at least 4 weeks. Forty-three geographically matched healthy individuals (21 females and 22 males; age range 18–71 years, median 45 years) were recruited in the healthy control group, with platelet counts ranging between 153 × 10^9^ and 294 × 10^9^/L (Table 1). This study was approved by the Medical Ethical Committee of Qilu Hospital, Shandong University, and conducted in accordance with the Declaration of Helsinki.

**Table 1:** Baseline characteristics of ITP patients and healthy controls.

	**ITP patients (*N* = 65)**	**Healthy controls (*N* = 43)**	***P***
Age	47 (16–81)	45 (18–71)	0.697
Gender			
Female	34 (52.30)	21 (48.84)	0.724
Male	31 (47.69)	22 (51.16)	0.724
Baseline platelet count (10^9^ per L)	19 (1–62)	194 (153–294)	<0.0001
ITP duration (months)	5 (1–240)	NA	NA
Previous therapies, *n*			
≤2	38 (58.46)	NA	NA
≥3	27 (41.53)	NA	NA

*Data are median (range) or n (%). NA, not applicable*.

### Decitabine

Decitabine (Sigma, Saint Louis, MO, USA) was dissolved in water for *in vitro* and animal studies. Decitabine solution was prepared freshly for each use.

### Murine Model

We established the murine ITP model by transferring 5 × 10^4^ splenocytes of C57BL/6 CD61 (B6.129S2-Itgb3tm1Hyn/JSemJ; Stock No: 008819) knockout mice, which were immunized against platelets weekly from syngeneic wild-type C57BL/6 mice (male, 8–12 weeks old, Center for New Drug Evaluation of Shandong University, Shandong, China) into irradiated severe combined immunodeficient (SCID) mice with a C57BL/6 background (J001913, 6–8 weeks old, Jackson Laboratory, Bar Harbor, ME) (23, 24). The successfully constructed murine model exhibited profound thrombocytopenia for 28–35 days after splenocyte transplant. To verify the effect of decitabine, low-dose (0.03 mg/kg) of decitabine or PBS were administrated intravenously three times a week from day 7. For PD-1 blockade assay, mice were treated with PBS, decitabine (0.03 mg/kg), anti-mouse PD-1 antibody (250 μg, RPM114, BioXcell), or decitabine plus anti-mouse PD-1 antibody. SCID mice were euthanatized on day 28 and splenocytes were incubated with fluorophore-conjugated monoclonal antibodies and analyzed by flow cytometry. All mouse experiments were approved by the Animal Care and Use Committee of Qilu Hospital and were conducted under the guidelines for Animal Care and Use of Shandong University, China.

### Isolation and Culturing of CD8^+^ T Cells

PBMCs were isolated from peripheral venous blood of ITP patients and healthy controls with Ficoll–Hypaque centrifugation (Amersham Biosciences, Piscataway, NJ, USA). CD8^+^ T cells were sorted from PBMCs by magnetic beads and MS columns (Miltenyi Biotec, Bergisch Gladbach, Germany) with a purity of over 90%.

For *in vitro* assays, CD8^+^ T cells were cultured in RPMI 1640 medium (Life Technologies, Paisley, UK) supplemented with 10% heat-inactivated fetal bovine serum (Gibco, Grand Island, NY, USA) and 1% penicillin and streptomycin (Solarbio, Beijing, China), and were stimulated with recombinant human IL-2 (5 ng/mL, R&D Systems, Minneapolis, MN, USA), anti-human CD3 antibodies (1 ng/mL, eBioscience, San Diego, CA, USA), and anti-human CD28 antibodies (1 ng/mL, eBioscience) for 72 h (37°C, 5% CO_2_). Recombinant fusion protein PD-L1-Fc (0.5 μg/mL, R&D Systems), decitabine (100 nM), anti-human PD-1 antibody (10 μg/mL, BioLegend, CA, USA), or PBS were added to the incubation media. Subsequently, CD8^+^ T cells were collected for flow cytometry, CTLs-induced platelet apoptosis, and bisulfite sequence PCR.

For ITP patients receiving decitabine, we isolated CD8^+^ T cells before (0) and after (12 weeks) the administration of decitabine and determine the function of CD8^+^ T cells by flow cytometry and CTLs-induced platelet apoptosis analysis.

### Quantitative Real-Time PCR

Total RNA was purified using TRIzol (Invitrogen, Grand Island, NY) and converted to cDNA using the PrimeScript RT reagent kit (Perfect Real Time; Takara). The mRNA expression of PD-1 (forward: 5′-CTCAGGGTGACAGAGAGAAG-3′; reverse: 5′-GACACCAACCAC- CAGGGTTT-3′) and endogenous control GAPDH (forward: 5′-GCACCGTCAAGGCTGAGAAC-3′; reverse: 5′-TGGTGAAGACGCCAGTGGA-3′) was quantified by real-time PCR using a LightCycler 480 System (Roche Applied Science, Mannheim, Germany).

### Flow Cytometry

For the comparison of ITP patients and healthy volunteers, we adjusted peripheral blood leukocyte suspension to 1 × 10^6^ cells/mL after removing the red blood cells by ACK lysing buffer. For the analysis of ITP murine models, we prepared single-cell suspension with a concentration of 1 × 10^6^ cells/mL by pulverizing murine spleens. *In vitro*, a total of 1 × 10^6^ CD8^+^ T cells were harvested from 24-well plates after incubation with 100 nM decitabine or PBS. To determine the expression of PD-1 and PD-L1, all suspensions were subsequently stained by anti-human or anti-mouse conjugated-antibodies (eBioscience) following the manufacturers' instructions. Cells were subsequently analyzed on a Gallios Flow cytometer with Kaluza Flow Cytometry Analysis Software (Beckman Coulter, Brea, CA, USA).

### CTLs-Induced Platelet Apoptosis

As previously described (7), CD8^+^ T cells isolated from patients receiving low-dose decitabine treatment were used as effector cells. Platelets of healthy donors were prepared by differential centrifugation and used as target cells. The concentrations of CD8^+^ T cells and platelets were adjusted to 10^5^ and 10^6^/mL, respectively. After co-culturing for 4 h, the cells were harvested to analyze the platelet apoptosis. Platelets were stained with JC-1 using mitochondrial membrane potential assay kit (Beyotime, Nantong, China). JC-1 aggregates exhibiting red fluorescence represented the live platelets and JC-1 monomer showing green fluorescence represented the apoptotic platelets. The percentage of JC-1 monomer on CD61 platelet was defined as the apoptosis rate. The level of spontaneous platelet apoptosis was determined in the tubes holding only culture media and platelets. CTL-induced platelet apoptosis was defined as the percentage of apoptosis in the tubes with CTLs and platelets minus the spontaneous platelet apoptosis. To further evaluate the effects on CTLs-mediated platelet apoptosis in ITP, recombinant fusion protein PD-L1-Fc, decitabine, anti-human PD-1 antibody, or PBS were applied to the culture system of platelets or platelets plus CD8^+^ T cells harvested after 72 h culturing *in vitro*. After co-culturing for 4 h, platelet apoptosis was measured.

### DNA Methylation Analysis

For PD-1 DNA methylation analysis, total DNA was extracted from CD8^+^ T cells by QIAamp DNA Mini Kit (Qiagen, Hilden, Germany). Complete bisulfite conversion of DNA was performed by EpiTect Bisulfite Kit (Qiagen, Hilden, Germany) according to the manufacturer's instructions. Methylation of PD-1 CpG island was subsequently evaluated using a bisulfite-sequencing PCR (BSP). The 340-bp region (−975 to −636) of CpG rich area in PD-1 promoter upstream of its transcription start site (TSS) was amplified. PCR products were gel purified and cloned using the pMD18-T (Takara) for Sequencing (Life Technologies, CA, USA). Ten individual clones of each sample were sequenced on an ABI 3730 XL DNA Analyzer and the resulting sequences were evaluated for methylated CpG sites using QUMA (quantification tool for methylation analysis, http://quma.cdb.riken.jp/). Primer sequences used for BSP of PD-1 promoter were designed as follows: forward, GGTTATTTTAAGTTGATGAGTTTG and reverse, CACACCATAACCACAATTCC.

### Western Blots Analysis

The downstream of PD-1 signaling included attenuating the activation of phosphatidylinositol 3-kinase (PI3K)/AKT pathway which can improve T lymphocyte metabolism, regulate cell cycle and apoptosis, and affect T lymphocyte activation and immune function (25–27), the protein expression of PI3K and AKT was analyzed. CD8^+^ T cells cultured with 100 nM decitabine or PBS were lysed in ice-cold RIPA buffer containing a protease inhibitor cocktail (BestBio, Shanghai, China) and concentrations of the protein were determined by BCA assay kit (BestBio). After denatured at 95°C for 5 min, the protein samples were loaded and separated by SDS-PAGE and transferred onto a NC membrane. Non-specific binding sites were blocked with 5% (w/v) fetal bovine serum at room temperature for 2 h. The membrane was incubated with polyclonal rabbit anti-human β-Actin (Cell Signaling Technology, NJ, USA), polyclonal rabbit anti-human PI3K, p-PI3K, AKT, and p-AKT antibodies (Cell Signaling Technology, NJ, USA). The blots were further incubated with HRP-conjugated secondary antibodies and washed three times in PBST for 5 min each time. Protein expression was detected by ECL and photographed by Bio-Spectrum Gel Imaging System (UVP, USA).

### Statistical Analysis

The results were expressed as median (IQR) or mean ± SD. Variables were investigated using the Shapiro-Wilk tests for normal distribution. Student's *t*-tests and ANOVA were used to analyze differences between the independent groups, and paired *t*-tests were performed for paired samples, unless the data were not normally distributed, in which case the Wilcoxon matched-pairs test and Mann-Whitney test were used. Categorical variables were compared using χ^2^ or Fisher's exact test. All tests were performed by Graphpad 8.0 system. *P* < 0.05 was considered statistically significant.

## Results

### Decreased PD-1 and PD-L1 Expression on CD8^+^ T Cells From ITP Patients

**Figure 1:**
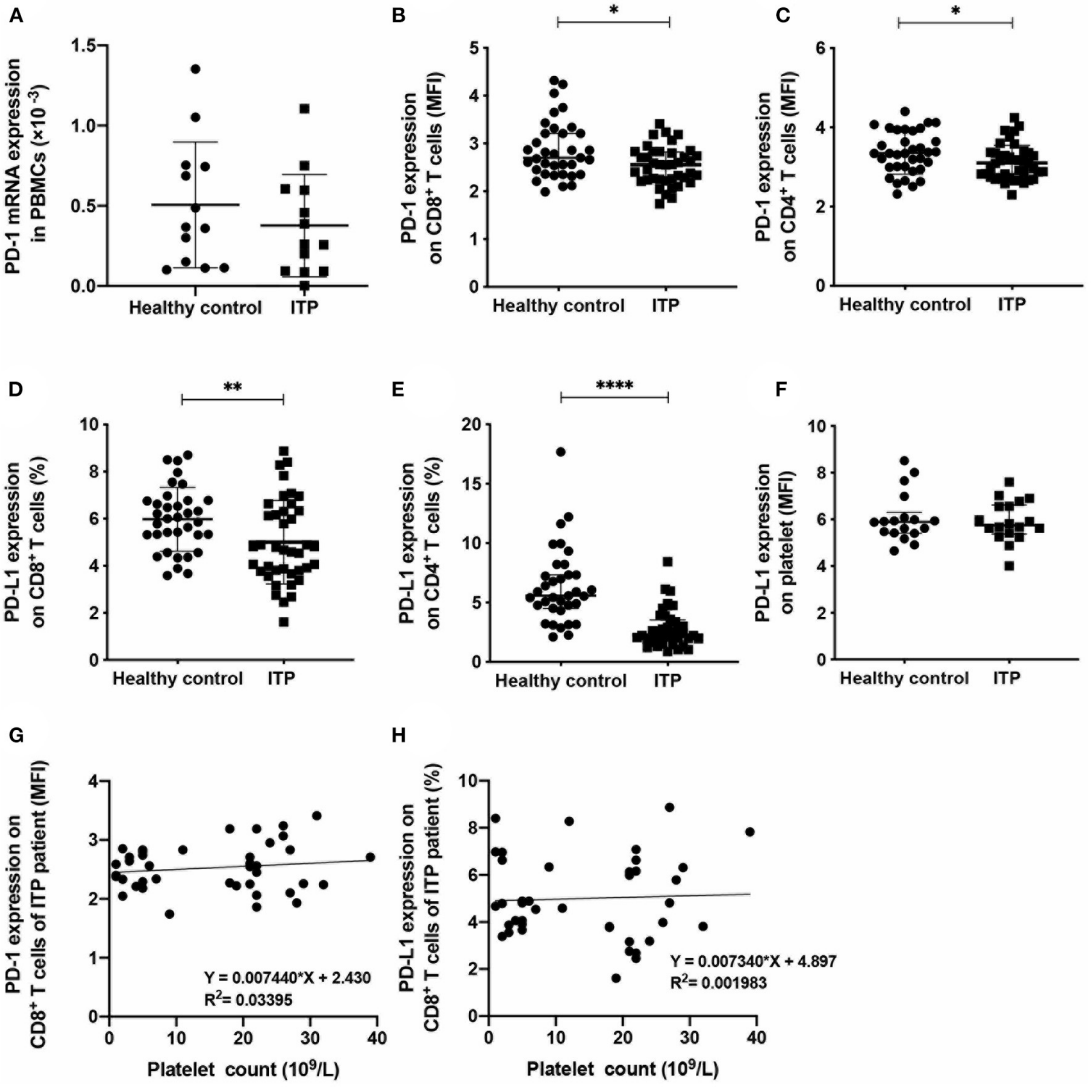
The expression of programed cell death protein 1 (PD-1) and PD-1 ligand (PD-L1) in immune thrombocytopenia (ITP) patients was lower than that of healthy controls. **(A)** The mRNA expression level of PD-1 in PBMCs from ITP patients had no difference with that of healthy controls (0.51 × 10^−3^ ± 0.39 × 10^−3^ vs. 0.37 × 10^−3^ ± 0.31 × 10^−3^, *n* = 13, *P* = 0.37, Unpaired *t*-test). **(B,C)** Significantly decreased PD-1 expression on CD8^+^ T cells (**B**, median; 2.70, IQR: 2.36–3.21, *n* = 35, vs. median: 2.56 IQR: 2.24–2.82, *n* = 40, *P* = 0.023, Mann Whitney test) and CD4^+^ T cells (**C**, 3.35 ± 0.53, *n* = 35 vs. 3.10 ± 0.45, *n* = 40, *P* = 0.032, Unpaired *t*-test) of ITP patients compared with healthy controls were observed. **(D,E)** Decreased PD-L1 expression on CD8^+^ T cells (**D**, 5.98 ± 1.35, *n* = 35 vs. 5.00 ± 1.77, *n* = 40, *P* = 0.010, Unpaired *t*-test) and CD4^+^ T cells (**E**, median: 5.58, IQR: 4.50–7.33, *n* = 35, vs. median: 2.25, IQR: 1.77–3.54, *n* = 40, *P* < 0.0001, Mann Whitney test) of ITP patients compared with healthy controls were observed. **(F)** The expression level of PD-L1 on platelet from ITP patients had no difference with that of healthy controls (median: 5.90, IQR: 5.42–6.31, *n* = 18, vs. median: 5.74, IQR: 5.34–6.61, *n* = 18, *P* = 0.894, Mann Whitney test). **(G,H)** Linear regression analysis of the PD-1 **(G)** and PD-L1 **(H)** expression on CD8^+^ T cells and platelet counts in ITP patients. *P < 0.05, **P < 0.01, ****P < 0.0001.

The mRNA level of PD-1 in PBMCs from ITP patients had no difference with that of healthy controls (Figure 1A). To determine the protein expression of PD-1 and PD-L1 on T cells, flow cytometry was performed. The expression of PD-1 on CD8^+^ T cells (Figure 1B) and CD4^+^ T cells (Figure 1C) in peripheral blood of ITP patients was significantly lower than that in healthy controls. We further tested PD-L1 expression on T cells. ITP patients expressed significantly lower PD-L1 on CD8^+^ T cells (Figure 1D) and CD4^+^ T cells (Figure 1E) compared with healthy controls. The expression level of PD-L1 on platelets from ITP patients had no difference with that of healthy controls (Figure 1F). In addition, the PD-1 (Figure 1G), and PD-L1 (Figure 1H) expression on CD8^+^ T cells was not associated with the platelet counts in ITP patients.

### PD-L1-Fc Inhibited CTLs-Mediated Platelet Apoptosis in ITP *in vitro*

**Figure 2:**
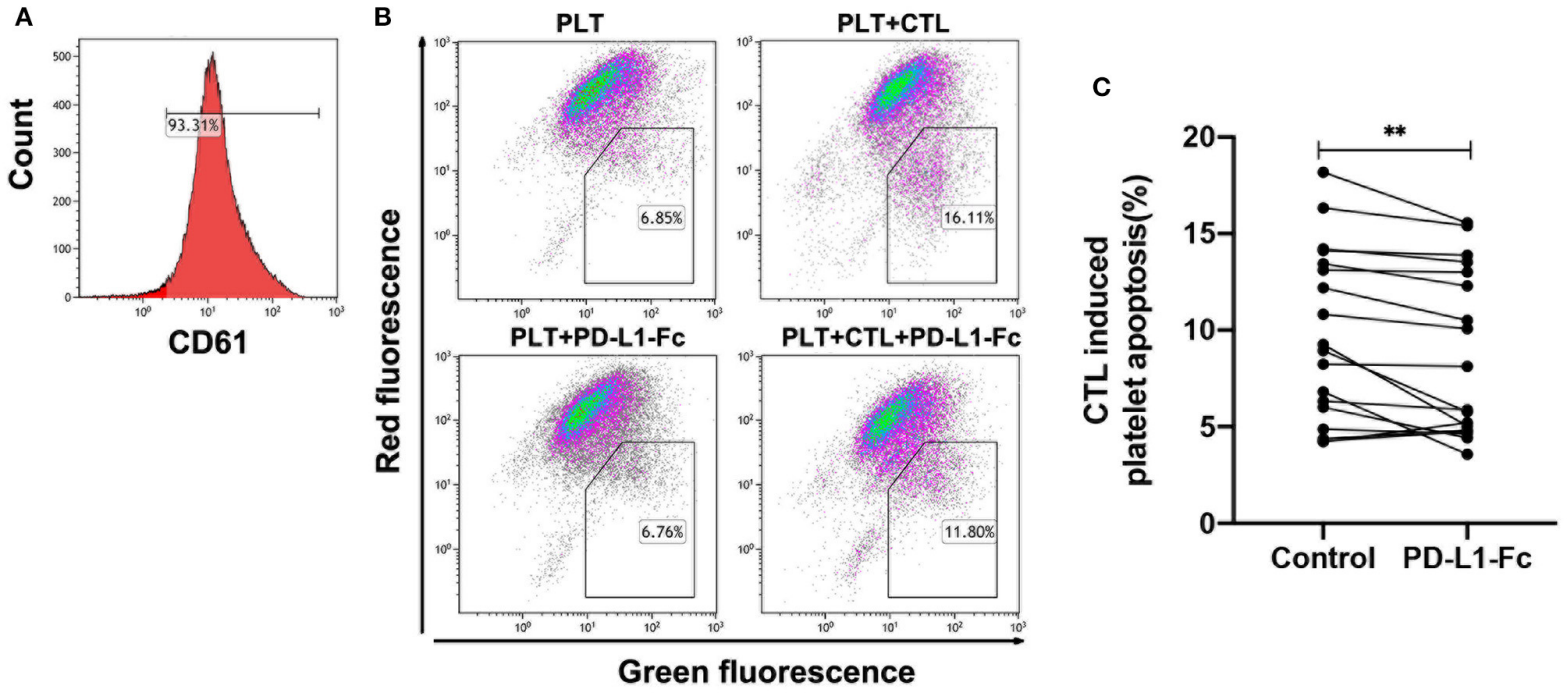
PD-L1-Fc reduced CTLs-mediated platelet apoptosis in ITP. **(A)** Platelets were gated by CD61 positive. **(B)** The gated dot-plots presented the apoptosis of platelets treated with PBS or PD-L1-Fc and cultured with CTLs of ITP patients treated with PBS or PD-L1-Fc. **(C)** A significant reduction was observed in CTLs-induced platelet apoptosis from ITP patients cultured with PD-L1-Fc *in vitro* after 72 h (9.76 ± 4.45 vs. 8.69 ± 4.29, *n* = 18, *P* = 0.006, Paired *t*-test). **P < 0.01.

To investigate the effect of PD-L1-Fc on CD8^+^ T cells in ITP, CTLs-mediated platelet apoptosis was analyzed to determine the cytotoxicity of CD8^+^ T cells after 72 h incubation. Compared with controls, PD-L1-Fc significantly decreased CTLs-mediated platelet apoptosis in ITP *in vitro* (Figure 2). PD-L1-Fc itself had no effect on platelet apoptosis.

### Increased Methylation of PD-1 in CD8^+^ T Cells From ITP Patients

**Figure 3:**
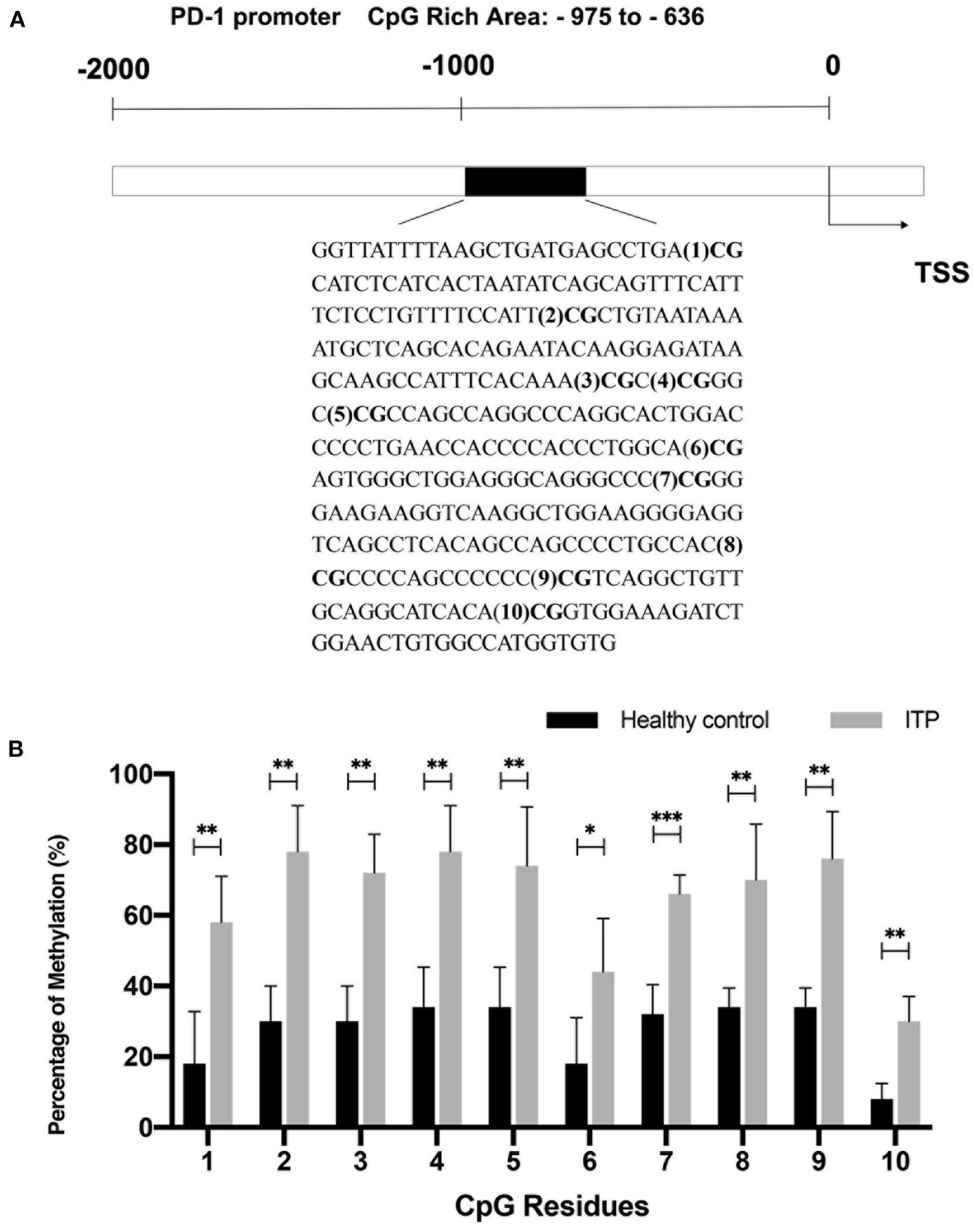
Increased PD-1 methylation in ITP patients. **(A)** Bisulfite sequencing PCR analysis of PD-1 methylation. The 340-bp region (−975 to −636) containing 10 CpG residues in CpG rich area upstream of transcriptional start site (TSS) was amplified and sequenced. **(B)** The percentage of methylated residues in the PD-1 promoter of CD8^+^ T cells in ITP patients was significantly higher compared with healthy controls (position 1, 18.00 ± 14.83% vs. 58.00 ± 13.04%, *P* = 0.006; position 2, 30.00 ± 10.00% vs. 78.00 ± 13.04%, *P* = 0.002; position 3, 30.00 ± 10.00% vs. 72.00 ± 10.95%, *P* = 0.002; position 4, 34.00 ± 11.40% vs. 78.00 ± 13.04%, *P* = 0.002; position 5, 34.00 ± 16.73% vs. 74.00 ± 11.40%, *P* = 0.006; position 6, 18.00 ± 13.04% vs. 44.00 ± 15.17%, *P* = 0.02; position 7, 32.00 ± 8.37% vs. 66.00 ± 5.48%, *P* = 0.0006; position 8, 34.00 ± 5.48% vs. 70.00 ± 15.81%, *P* = 0.005; position 9, 34.00 ± 5.58% vs. 76.00 ± 13.42%, *P* = 0.002; position 10, 8.00 ± 4.47% vs. 30.00 ± 7.07%, *P* = 0.002, *n* = 5, Multiple *t*-tests). *P < 0.05, **P < 0.01, ***P < 0.001.

We evaluated the promoter methylation level of PD-1 in CD8^+^ T cells from ITP patients and healthy controls. The 340-bp region (−975 to −636) containing 10 CpG residues in CpG rich area up-stream of TSS was amplified and sequenced based on BSP (Figure 3A). We found that the percentage of methylated residues in PD-1 promoter was significantly higher in ITP patients than that in controls (Figure 3B), suggesting a significantly higher PD-1 methylation level in ITP patients.

### Low-Dose Decitabine Restored PD-1 Expression on CD8^+^ T Cells and Ameliorated Thrombocytopenia in ITP

**Figure 4:**
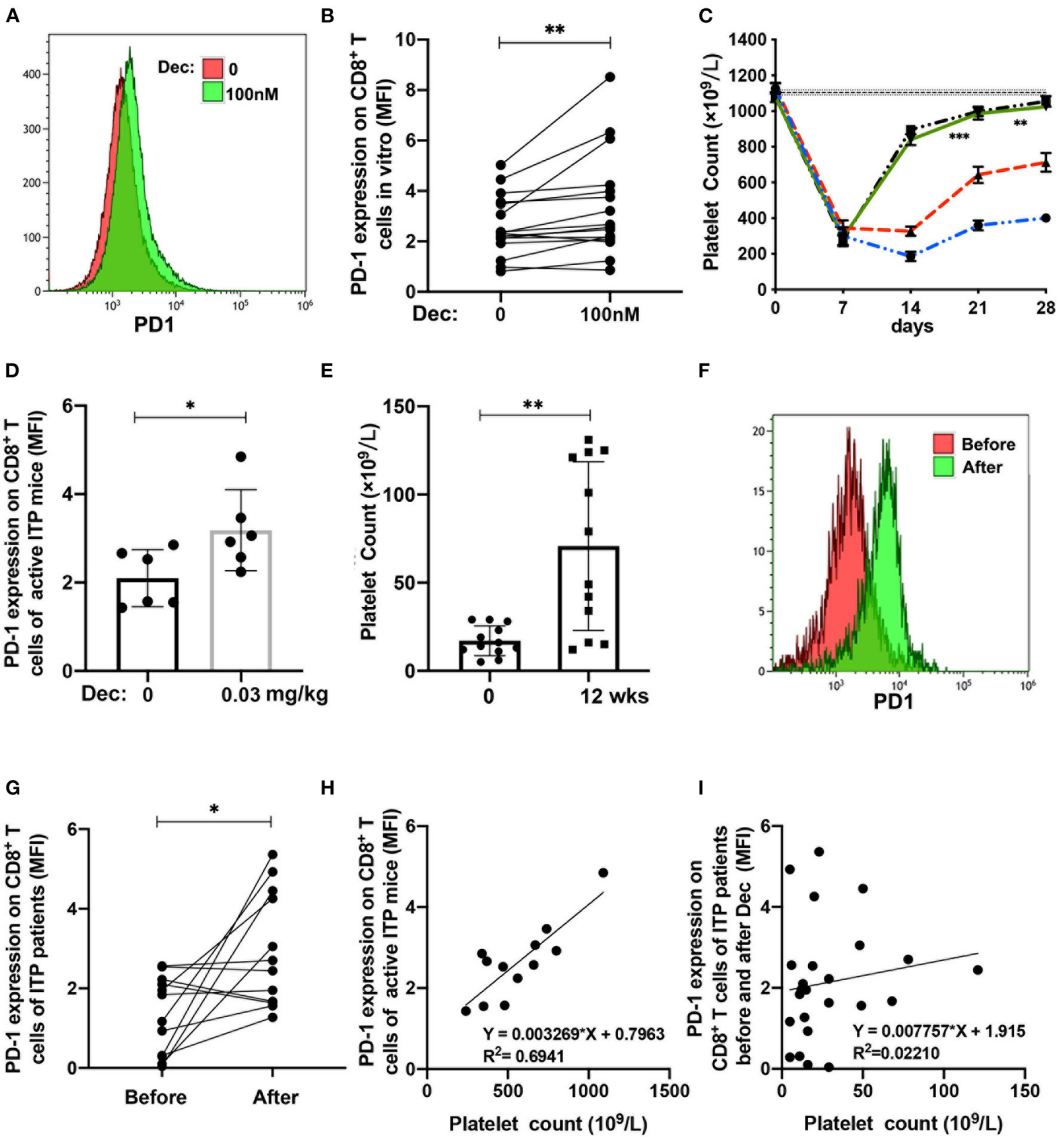
Low-dose decitabine (Dec) increased PD-1 expression on CD8^+^ T cells and ameliorated thrombocytopenia in ITP. **(A,B)** Low-dose decitabine significantly increased PD-1 expression on CD8^+^ T cells *in vitro* (**B**, median: 2.33, IQR: 1.98–3.54 vs. median: 2.61, IQR: 2.09–4.18, *n* = 16, *P* = 0.001, Wilcoxon test). **(C)** Platelet counts in severe combined immunodeficiency (SCID) mice that received 5 × 10^4^ splenocytes from either wild type C57 mice (♦, *n* = 10) treated with an equal volume of PBS, non-immunized CD61-knockout (KO) mice (▾, *n* = 10) treated with an equal volume of PBS, or platelet-immunized CD61-KO mice treated with an equal volume of PBS (•, *n* = 10) or decitabine (▴, *n* = 10). On day 21 and 28, a significantly higher platelet count was observed in the decitabine-treated group compared with PBS (day 21, 360.0 × 10^9^ ± 27.04 × 10^9^/L vs. 643.0 × 10^9^ ± 45.97 × 10^9^/L; *P* = 0.0005; day 28, 401.0 × 10^9^ ± 23.87 × 10^9^/L vs. 713.0 × 10^9^ ± 52.35 × 10^9^/L; *P* = 0.001). The horizontally dotted lines represent the normal mean platelet count ± SEM (dotted lines) from 40 SCID mice. Significance among groups was determined by ANOVA. **(D)** The expression of PD-1 on CD8^+^ T cells were upregulated in the spleen in decitabine treated ITP mice (**D**, 2.10 ± 0.65 vs. 3.18 ± 0.92, *n* = 6, *P* = 0.039, Unpaired *t*-test). **(E)** Increased platelet count was observed after low-dose decitabine treatment in ITP patients (17.08 × 10^9^ ± 8.53 × 10^9^/L vs. 70.75 × 10^9^ ± 47.79 × 10^9^/L, *n* = 12, *P* = 0.004, Paired *t*-test). **(F,G)** Elevated PD-1 expression on CD8^+^ T cells was observed after low-dose decitabine treatment in ITP patients (**G**, 1.24 ± 1.00 vs. 3.62 ± 2.28, *n* = 12, *P* = 0.018, Paired *t*-test). **(H)** Linear regression analysis of PD-1 expression and platelet counts in active ITP mice on day 28 after decitabine therapy (PBS group and decitabine group). **(I)** Linear regression analysis of the PD-1 expression on CD8^+^ T cells and platelet counts in ITP patients before and after decitabine treatment. *P < 0.05, **P < 0.01, ***P < 0.001.

To assess whether low-dose decitabine could modulate CTLs activity in ITP patients, we isolated CD8^+^ T cells from ITP patients and cultured with PBS or 100 nM decitabine. Low-dose decitabine significantly increased PD-1 expression on CD8^+^ T cells from ITP patients *in vitro* (Figures 4A,B). To assess whether our *in vitro* observations were reproducible *in vivo*, we adopted an active ITP murine model. Following the irradiation and transfer of anti-CD61 immune-sensitized splenocytes into SCID mice, platelet counts dropped to nadir on day 7. On day 21 and 28, significantly higher platelet counts were observed in the decitabine-treated group compared to control group (Figure 4C). We quantified the expression of PD-1 in the active murine ITP models and ITP patients receiving decitabine administration. Compared with controls, PD-1 was increased on splenic CD8^+^ T cells in decitabine-treated ITP mice (Figure 4D). Furthermore, a significant increase in platelet counts (Figure 4E) and in PD-1 expression on CD8^+^ T cells in ITP patients was observed after decitabine treatment (Figures 4F,G). We analyzed correlation between PD-1 expression on CD8^+^ T cells and platelet counts in active ITP murine models. Taking PBS group and decitabine group together, the PD-1 expression on CD8^+^ T cells was associated with the platelet counts in active ITP mice on day 28 (Figure 4H). For ITP patients, the PD-1 expression on CD8^+^ T cells was not related to the platelet counts in ITP patients before and after decitabine treatment (Figure 4I).

### Low-Dose Decitabine Decreased PD-1 Promoter Methylation in ITP

**Figure 5:**
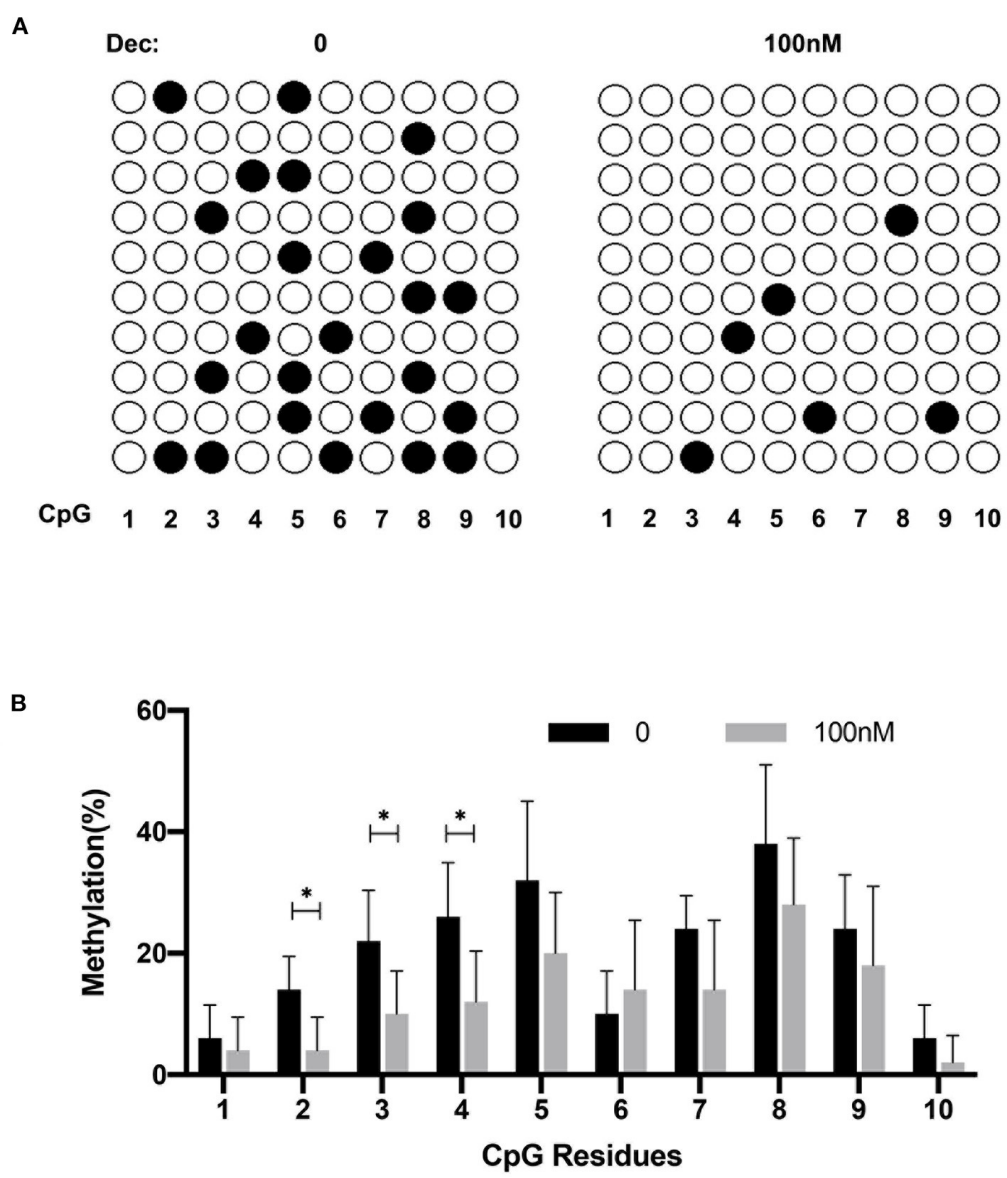
Low-dose decitabine (Dec) decreased PD-1 promoter methylation in ITP *in vitro*. **(A)** The representative plots of bisulfite sequencing analysis of PD-1 methylation. **(B)** Low-dose decitabine significantly decreased the percentage of methylated CpG residues of PD-1 in CD8^+^ T cells (position 2, 14.00 ± 5.48% vs. 4.00 ± 5.48%, *P* = 0.020; position 3, 22.00 ± 8.37% vs. 10.00 ± 7.07%, *P* = 0.040, position 3, 26.00 ± 8.94% vs. 12.00 ± 8.47%, *P* = 0.034, *n* = 5, Multiple *t*-test). *P < 0.05.

CD8^+^ T cells from ITP patients were cultured with 100 nM decitabine or PBS. BSP was performed to detect PD-1 gene methylation. Treated cells showed significantly lower methylated CpG residues compared with controls (Figure 5).

### Low-Dose Decitabine Inhibited CTLs-Mediated Platelet Apoptosis in ITP

**Figure 6:**
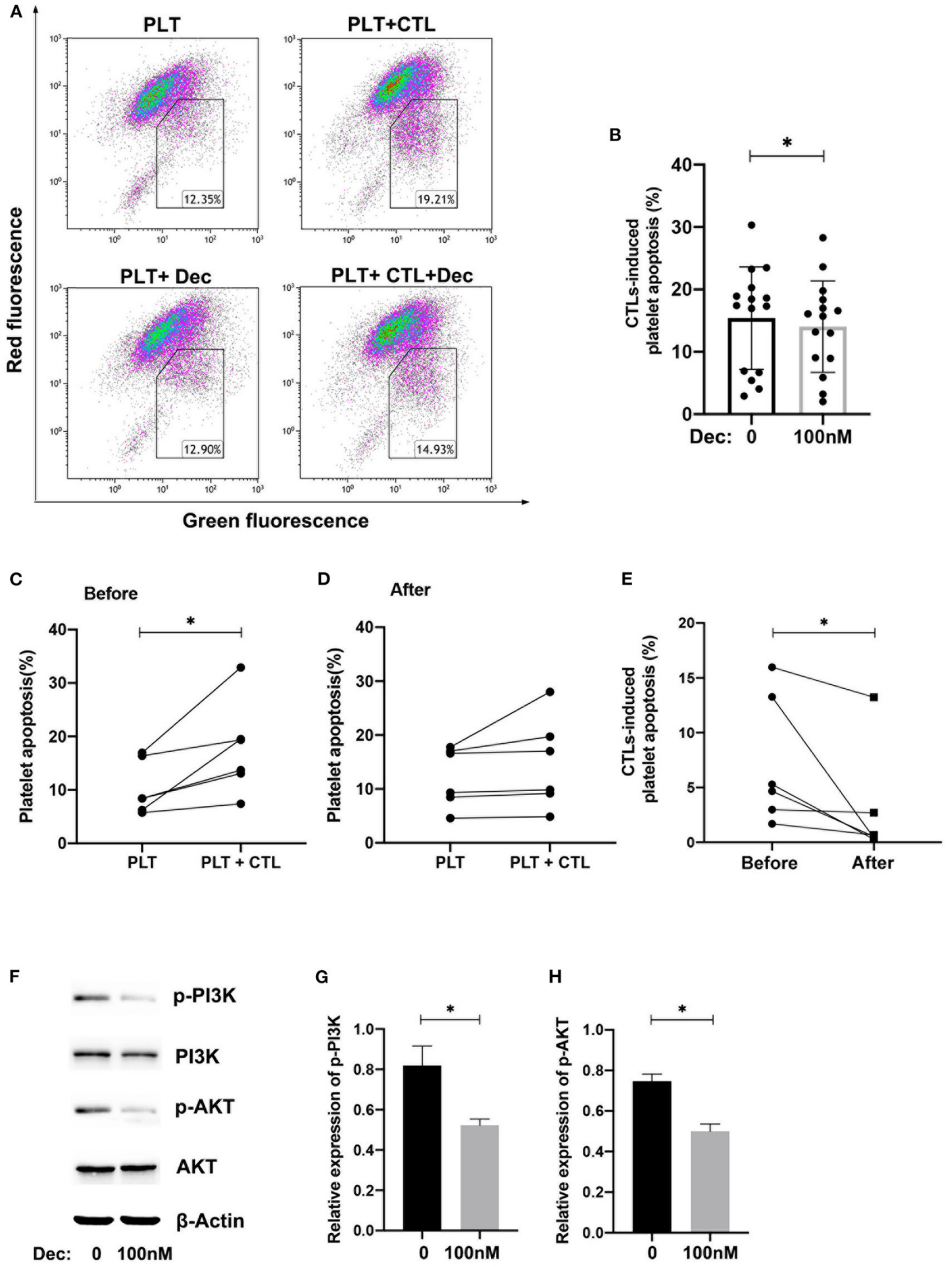
Low-dose decitabine (Dec) inhibited CTLs-mediated platelet apoptosis in ITP. **(A)** The representative dot-plots characterized the apoptosis of platelets treated with PBS or 100 nM decitabine and cultured with CTLs of ITP patients treated with PBS or 100 nM decitabine. **(B)** A significant reduction in CTLs induced apoptosis of platelets cultured with low-dose decitabine was observed (15.40 ± 8.23 vs. 14.05 ± 7.32, *n* = 15, *P* = 0.018, Paired *t*-test). **(C–E)** Platelet spontaneous apoptosis and platelet apoptosis cultured with CTLs was measured before **(C)** and after **(D)** 12-week decitabine treatment for ITP patients. **(E)** CTLs-induced platelet apoptosis was significantly inhibited after 12-week decitabine administration (before: median: 4.98, IQR: 2.66–13.95 vs. after: median: 0.61, IQR: 0.37–5.34, *n* = 6, *P* = 0.031, Wilcoxon test). **(F)** Isolated CD8^+^ T cells were treated with PBS control or decitabine. Western blotting was performed on cell lysates. **(G,H)** Graphs present densitometry data of relative phosphorylated (p-) protein to total protein after PBS or 100 nM decitabine treatment (**G**, p-PI3K, 0.82 ± 0.095 vs. 0.53 ± 0.03, *P* = 0.017; **H**, p-AKT, 0.75 ± 0.035 vs. 0.50 ± 0.036, *n* = 3, *P* = 0.035, Paired *t*-test). *P < 0.05.

To study the effect of decitabine on CTLs cytotoxicity, we isolated CD8^+^ T cells from ITP patients and cultured with PBS or 100 nM decitabine *in vitro* (Figure 6A). A significant reduction in CTLs induced apoptosis of platelets co-cultured with low-dose decitabine was observed (Figure 6B). Further, we investigated CTLs cytotoxicity of ITP patients receiving decitabine treatment and found that CTLs induced platelet apoptosis was significantly inhibited after 12-week decitabine administration (Figures 6C–E).

### Low-Dose Decitabine Inhibited the Phosphorylation of PI3K and AKT

Western blot analysis of cell lysates from CD8^+^ T cells cultured with 100 nM decitabine revealed that there was no significant change in the expression levels of PI3K and AKT. However, the phosphorylation levels of PI3K and AKT were significantly downregulated by low-dose decitabine (Figure 6F). Similarly, the ratio of p-PI3K/PI3K and p-AKT/AKT also showed a significant downregulation (Figures 6G,H). These results indicated that low-dose decitabine has an inhibitory effect on PI3K/AKT signaling pathway in CD8^+^ T cells.

### Blocking PD-1 Partly Counteracted the Therapeutic Effect of Low-Dose Decitabine in Inhibiting CTLs-Mediated Platelet Apoptosis in ITP

**Figure 7:**
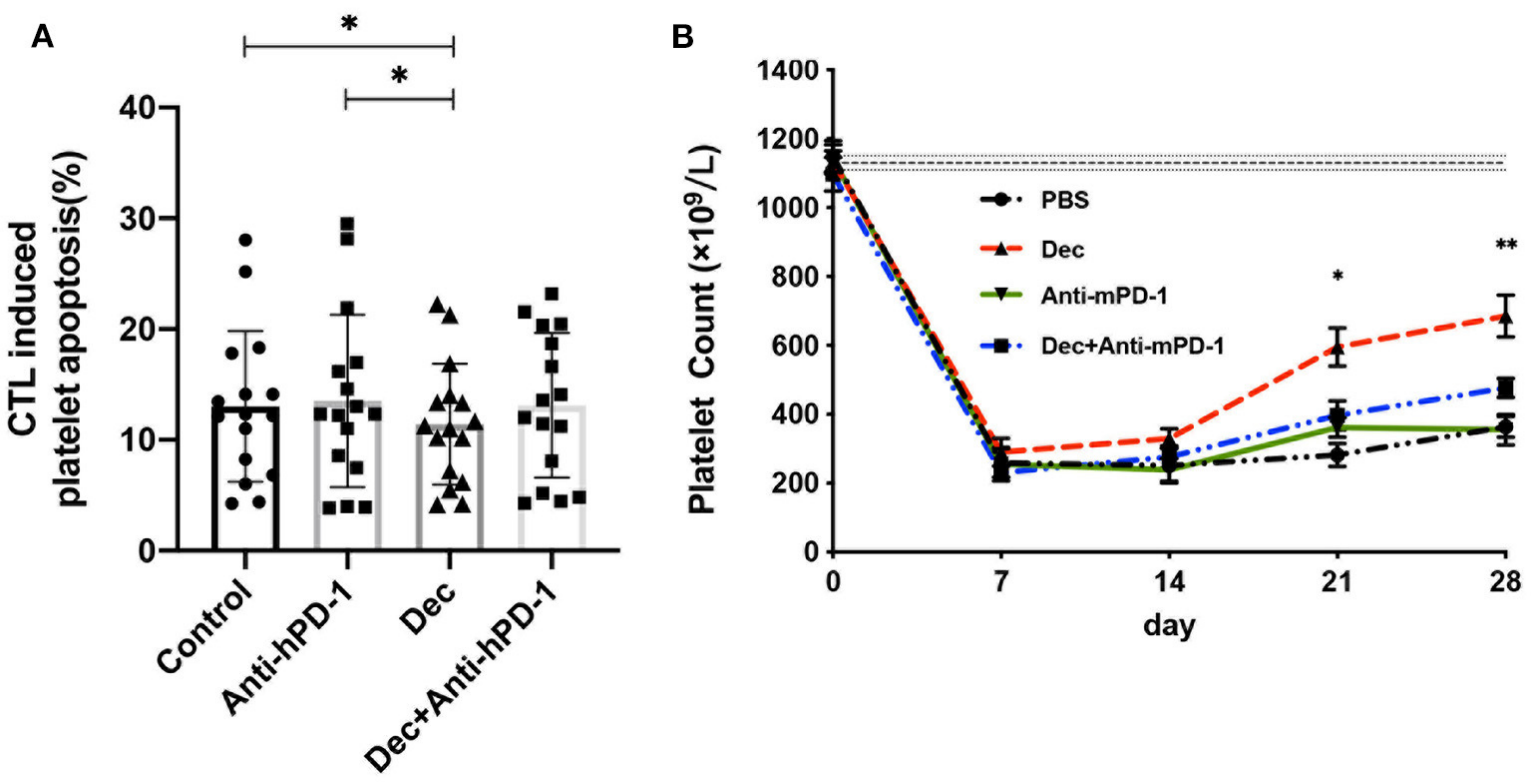
Blocking PD-1 can partly counteract the therapeutic effect of low-dose decitabine in inhibiting CTLs-mediated platelet apoptosis in ITP *in vitro* and *in vivo*. **(A)** Anti-human PD-1 antibody (Anti-hPD-1) partly counteracted decitabine effect in inhibiting CTLs-induced platelets apoptosis in ITP *in vitro* (13.63 ± 8.05 at Anti-hPD-1 vs. 13.12 ± 6.53 at Dec + Anti-hPD-1, *n* = 16, *P* = 0.961; 13.63 ± 8.05 at Anti-hPD-1 vs. 11.86 ± 6.48 at Dec, *n* = 16, *P* = 0.043, ANOVA). **(B)** Anti-mouse PD-1 antibody (Anti-mPD-1) offset the low-dose decitabine treatment in active ITP mice on days 21 and 28 (day 21, 595.0 × 10^9^ ± 55.42 × 10^9^/L at Dec vs. 396.7 × 10^9^ ± 42.40 × 10^9^/L at Dec + Anti-mPD-1, *P* = 0.026; day 28, 685.0 × 10^9^ ± 51.03 × 10^9^/L at Dec vs. 476.7 × 10^9^ ± 27.16 × 10^9^/L at Dec + Anti-mPD-1, *n* = 6, *P* = 0.008, ANOVA). *P < 0.05, **P < 0.01.

To determine whether the mechanism of decitabine was dependent on PD-1 pathway or not, we performed assays for PD-1 blockade test *in vitro*. CD8^+^ T cells from ITP patients were divided into four groups, including control, anti-human PD-1 antibody (Anti-hPD-1), 100 nM decitabine, and anti-hPD-1 combined with 100 nM decitabine. As shown in Figure 7A, there was no difference in CTLs induced platelet apoptosis among anti-hPD-1 group, 100 nM decitabine group plus anti-hPD-1 group, or control group. A significant decrease was found in 100 nM decitabine group. To further evaluate the effect of PD-1 blockade *in vivo*, we applied anti-mouse PD-1 antibody (Anti-mPD-1) in ITP murine model. ITP mice were divided into 4 groups: control, anti-mPD-1, decitabine, and decitabine combined with anti-mPD-1. Platelet counts in decitabine-treated ITP mice plus anti-mPD-1 were not different from those in anti-mPD-1 or control mice, but decreased significantly compared with decitabine-treated ITP mice on day 21 and on day 28 (Figure 7B). These results suggested that the inhibitory effect of decitabine was partly counteracted by blocking PD-1.

## Discussion

In the present study, our data indicated that the expression of PD-1 and PD-L1 on CD8^+^ T cells was decreased. Enhancing PD-1/PD-L1 signaling by PD-L1-Fc could reduce CTLs cytotoxicity. PD-1 promoter methylation in CD8^+^ T cells was increased in ITP patients compared with healthy controls. Low-dose decitabine decreased PD-1 promoter methylation, increased PD-1 expression, and inhibited CTLs-mediated platelet destruction in ITP. The inhibitory effect of decitabine on CTLs cytotoxicity was partially counteracted by blocking PD-1. In addition to PD-1 signaling pathway, several other factors have been proved to be involved in the cytotoxicity of CD8^+^ T cells to platelets. Previous studies have demonstrated that CD8^+^ T cells could exert their cytotoxicity toward platelets by causing direct lysis or apoptosis while co-cultured with platelets *in vitro* (28, 29). The cytotoxicity of CD8^+^ T cells to autologous platelets was dependent on the increased granzyme B activity in some ITP patients (7). Recently, platelet desialylation was reported to play important roles in CD8^+^ T cells mediated platelet clearance in the liver in ITP patients (30).

However, the pathophysiology governing the breakdown of immune tolerance to platelet antigens and CTLs activation in ITP remains unclear. Binding of PD-1 on T cells to its ligands PD-L1 acts as a negative regulator of T-cell activation and is a crucial factor responsible for maintaining peripheral tolerance. The PD-1/PD-L1 pathway is an important reason for immune escape of malignant tumors from CTLs (7, 31, 32). Recent studies showed that PD-1 genetic deficiency in mice resulted in spontaneous development of arthritis, dilated cardiomyopathy, or lupus-like glomerulonephritis (14, 33, 34). Blocking PD-1/PD-L1 pathway by a monoclonal antibody directed to PD-1 exacerbated the development of autoimmune disease in murine model, such as experimental autoimmune encephalomyelitis (EAE) (35) and diabetes (36), demonstrating that PD-1/PD-L1 pathway regulated autoimmunity by limiting the expansion and activation of autoreactive CD8^+^ T cells (37). PD-1 inhibitors, such as nivolumab and pembrolizumab, showed therapeutic benefits in the treatment of cancers, but aroused immune-related adverse side effects (38, 39). Previous studies reported that PD-1 and PD-L1 expression on PBMCs was abnormal in patients with ITP (16, 40). In our study, we found significantly decreased PD-1 and PD-L1 expression on CD4^+^ and CD8^+^ T cells in ITP patients, which may contribute to the over-activation of CTLs in ITP. Additionally, activating PD-1/PD-L1 pathway by PD-L1 Ig lessens autoantibody and inflammatory cytokine production in murine models of SLE and RA (41, 42). In ITP, activating PD-1/PD-L1 signal pathway by PD-L1-Fc promoted the death of CD4^+^ and CD8^+^ T cells, and decreased the secretion of IFN-γ and IL-2 (17). Consistently, our results demonstrated that PD-L1-Fc decreased CTLs-mediated platelet apoptosis. These findings indicated that stimulation of PD-1/PD-L1 signaling might be an effective therapeutic approach in ITP.

Epigenetic abnormalities involved in regulating gene expression without changing the nucleotide sequences were related to T cell activation and induced the occurrence and development of autoimmune diseases. Aberrant DNA and histone methylation were proved to participate in the pathogenesis of ITP (43, 44). Recent studies showed that DNA methyltransferase-3A (DNMT-3A) and DNMT-3B levels in adult ITP patients were significantly lower than those of normal subjects (45). We proposed that abnormal methylation contributed to the decreased PD-1 expression in ITP. In this study, PD-1 promoter hypermethylation is documented and to our knowledge, this is the first study demonstrating PD-1 methylation in ITP. Decitabine is a hypomethylating agent with a dual pharmacological action. Low-dose decitabine exhibited the demethylating effect inducing cell differentiation and maturation and high concentrations of decitabine presented cytotoxic activity (46, 47). Decitabine has been applied in the management of MDS and sickle cell disease. Previous studies demonstrated that potentially due to decitabine therapy, the PD-1 CpG island demethylated and PD-1 expression increased. As a result, the immune landscape in MDS may shift from a T cell-activated environment to an immune suppressive environment (21, 48). Low-dose decitabine could enhance tumor necrosis factor-related apoptosis-inducing ligand (TRAIL) expression by decreasing its promoter methylation status in megakaryocytes in ITP (19). In our study, low-dose decitabine decreased PD-1 promoter methylation and restored PD-1 expression. Intriguingly, the PD-1 expression on CD8^+^ T cells was associated with the platelet counts in active ITP mice after decitabine therapy, suggesting that the effect of low-dose decitabine in ameliorating thrombocytopenia in ITP might be related to the increasing PD-1 expression on CD8^+^ T cells. This correlation was not found in ITP patients receiving decitabine therapy, which might be due to the heterogeneity of ITP patients and a large sample size was required to reach a significant correlation. Further, our data showed that low-dose decitabine suppressed the cytotoxicity of CTLs in ITP, which could be neutralized by anti-PD-1 antibody.

The combination of PD-1 and PD-L1, along with T-cell receptor (TCR) signaling, leads to attenuation of the PI3K/AKT pathway, resulting in the inhibition of downstream signaling and further inducing CD8^+^ T and CD4^+^ T cell anergy (49). The PD-1/PD-L1 blockade therapy could decrease apoptosis in CD8^+^ T cells via the PI3K/AKT/mTOR signaling pathway (50). The cell proliferation-related signal transduction pathway PI3K/AKT is involved in the regulation of a variety of cell proliferation and plays a pivotal role in the regulation of autoimmunity, inflammation, and cancers. Previous studies have shown that the activation of PI3K/AKT/mTOR pathway can increase nutrient uptake and energy production in CD8^+^ T cells (51, 52). In ITP, the homeostasis of CD4^+^ T cells might be restored by indirubin via a PTEN/AKT/mTOR signaling pathway (16). In our *in vitro* study, low-dose decitabine was found to decrease the levels of p-PI3K and p-AKT, suggesting that the inhibitory effect of low-dose decitabine on CD8^+^ T cells might be related to PI3K/AKT signaling pathway in ITP.

In summary, our findings revealed that low-dose decitabine inhibited CTLs cytotoxicity via upregulation of PD-1 expression on CD8^+^ T cells and restoration of PD-1 methylation. This novel mechanism of low-dose decitabine underlies the potential therapeutic strategy for ITP.

## Data Availability Statement

The original contributions presented in the study are included in the article/supplementary material, further inquiries can be directed to the corresponding author/s.

## Ethics Statement

The studies involving human participants were reviewed and approved by Medical Ethical Committee of Qilu Hospital, Shandong University. The patients/participants provided their written informed consent to participate in this study. The animal study was reviewed and approved by Medical Ethical Committee of Qilu Hospital, Shandong University.

## Author Contributions

PH and HZ performed the experiment and wrote the manuscript. HZ and MH designed and supervised the research. YH, TY, YZ, YL, YS, HW, and PX assisted the experiment. GL, TS, XH, XL, LL, and JP analyzed the data. All authors read and approved the final manuscript.

## Conflict of Interest

The authors declare that the research was conducted in the absence of any commercial or financial relationships that could be construed as a potential conflict of interest.
